# Impact of Respiratory Bacterial Findings on Patients with Chronic Pulmonary Aspergillosis

**DOI:** 10.1155/2024/1329884

**Published:** 2024-04-30

**Authors:** Hyun Kyu Cho, Seong Mi Moon, Hyoung-Tae Kim, Beomsu Shin

**Affiliations:** ^1^Division of Pulmonary and Critical Care Medicine, Department of Medicine, Samsung Changwon Hospital, Sungkyunkwan University School of Medicine, Changwon, Republic of Korea; ^2^Division of Pulmonary and Critical Care Medicine, Department of Internal Medicine, Chungbuk National University Hospital, Chungbuk National University College of Medicine, Cheongju, Republic of Korea; ^3^Department of Laboratory Medicine, Samsung Changwon Hospital, Sungkyunkwan University School of Medicine, Changwon, Republic of Korea; ^4^Department of Allergy, Pulmonology and Critical Care Medicine, Gil Medical Center, Gachon University, Incheon, Republic of Korea

## Abstract

**Background:**

Distinct bacterial strains may affect the prognosis of patients with chronic respiratory diseases. However, little is known about the clinical significance of respiratory bacteria in patients with chronic pulmonary aspergillosis (CPA), a progressive and debilitating disease caused by *Aspergillus* spp.

**Objectives:**

This study aimed to analyze data obtained from CPA patients and their sputum or bronchial washing samples and investigate the prevalence and composition of respiratory bacteria and clinical implications. *Patients and Methods*. We retrospectively reviewed the data of patients diagnosed with CPA between March 2019 and February 2023 in a tertiary referral hospital. We assessed the clinical characteristics and overall and pneumonia-specific survival rates of patients with CPA based on the presence of bacteria. *Results and Conclusion*s. We included 142 patients with CPA. The most commonly identified bacteria were *Klebsiella pneumoniae* (22.5%), followed by *Pseudomonas aeruginosa* (21.8%) and *Escherichia coli* (4.2%). Patients with isolated bacteria had a higher prevalence of older age, female sex, diabetes, and a history of extrathoracic malignancy than those without isolated bacteria (*P* = 0.024, 0.013, 0.021, and 0.034, respectively). Furthermore, over a median follow-up of 11 (4–21) months, the pneumonia-specific mortality rate was 13.4% (19/142), which was higher in patients with isolated bacteria than in those without (*P* = 0.045, log-rank test). Particularly, patients with the presence of *P. aeruginosa* had a significantly higher mortality rate from pneumonia than those without the presence of *P. aeruginosa* (adjusted hazard ratio, 3.34; *P* = 0.015). In conclusion, CPA patients with isolated bacteria, especially *P. aeruginosa*, showed higher mortality rates due to pneumonia. Performing tests to identify bacteria in the lower respiratory tract of patients with CPA may be helpful in predicting future prognosis. Further studies are required to validate these findings in diverse ethnic groups.

## 1. Introduction

Chronic pulmonary aspergillosis (CPA) is a progressive fungal infection caused by *Aspergillus* species, usually most commonly caused by *Aspergillus fumigatus* [1]. It primarily affects patients with mild immunodeficiencies and pre-existing structural lung diseases, such as sequelae of pulmonary tuberculosis (PTB), bronchiectasis, nontuberculous mycobacterial pulmonary disease (NTM-PD), and emphysema [1]. CPA has various clinical presentations and phenotypes including simple aspergilloma, Aspergillus nodule, chronic cavitary pulmonary aspergillosis, and subacute invasive aspergillosis [2]. CPA has a high mortality rate of 50–80% over 5 years due to limited treatment options and the presence of comorbidities [3, 4]. Recurrent superinfection is an associated comorbidity that further complicates treatment plans and often leads to adverse outcomes, including mortality [5, 6].

Inflammatory chronic lung disease harbors different respiratory bacterial communities that can be associated with disease pathogenesis and prognosis [7, 8]. Patients with chronic respiratory diseases, such as bronchiectasis, chronic obstructive pulmonary disease (COPD), and interstitial lung disease (ILD), are chronically infected or colonized with potentially pathogenic microorganisms, resulting in a worse quality of life due to persistent respiratory symptoms, recurrent infection, or exacerbation [9–11]. CPA is usually accompanied by cavitary lesions in the lungs, which are often colonized by potentially pathogenic microorganisms, resulting in an increased infection of the lung parenchyma [2]. In previous studies, representative bacteria in CPA, similar to other chronic lung diseases, including *Streptococcus pneumoniae* (*S. pneumoniae*), *Haemophilus influenzae* (*H. influenzae*), *Staphylococcus aureus (S. aureus)*, *Pseudomonas aeruginosa* (*P. aeruginosa*) and anaerobic bacteria, were observed [12, 13]. Despite its potential impact on the progression of CPA, its role in the clinical course of bacterial isolation in patients with CPA remains poorly understood due to a lack of research [4, 12].

To address this knowledge gap, this study aimed to analyze data obtained from CPA patients and their sputum or bronchial washing samples and investigate the prevalence and composition of respiratory bacteria and clinical implications during the study period.

## 2. Materials and Methods

### 2.1. Study Population

**Figure 1:**
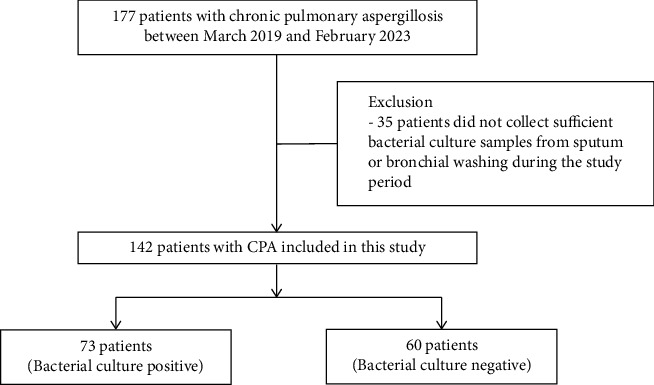
Patient flow chart.

We retrospectively reviewed the data of patients diagnosed with CPA at Samsung Changwon Hospital (a 761-bed tertiary referral hospital in Changwon, South Korea) between March 2019 and February 2023. A total of 177 patients with CPA were initially considered. Bacterial culture is usually performed when the physician suspects bacterial pneumonia or when patients complain of respiratory symptoms such as coughing up phlegm or hemoptysis. Among these, 35 were excluded because specimens for bacterial culture from sputum or bronchial washing were not obtained or were not suitable for analysis. In total, 142 patients were included in this study (Figure 1).

### 2.2. Ethics Statement

This study was approved by the Institutional Review Board of Samsung Changwon Hospital (IRB No. SCMC 2023-08-003). Due to the retrospective nature of the study, the requirement of informed consent was waived. The authors confirm that the ethical policies of the journal, as noted on the journal's author guideline page, have been followed, and appropriate approval has been received from the ethics review committee.

### 2.3. Diagnosis of CPA

During the study period, the diagnosis of CPA required a combination of clinical, radiological, and microbiological findings in the absence of an alternative diagnosis. Diagnostic criteria were (1) the presence of compatible chronic respiratory symptoms, including cough, sputum, dyspnea, or hemoptysis, lasting for at least 3 months; (2) the presence of compatible chest radiological findings, such as a cavity containing one or more aspergillomas or irregular intraluminal material with evidence of radiological progression (e.g., expansion of cavity size, new cavities, or increasing paracavitary infiltrates); and (3) positive serum anti-Aspergillus immunoglobulin G (IgG) antibodies (Aspergillus fumigatus IgG ELISA Kit; IBL International, Hamburg, Germany; or Aspergillus fumigatus IgG Fluorescent Enzyme Immonoassay Kit, ImmunoCAP Allergen m3 by Phadia ImmunoCAP 250; Thermo Fisher Scientific AB, Uppsala, Sweden) or isolation of Aspergillus species from respiratory specimens [1, 13]. Patients with simple aspergilloma, characterized by a fungal ball in the pulmonary or pleural cavity or ectatic bronchus without progression over months, and subacute invasive aspergillosis, defined as a nonchronic form of pulmonary aspergillosis that occurs in mildly immunocompromised patients, were excluded from this study [1]. All patients were assessed for cavitary lesions using a chest computed tomography (CT) scan.

### 2.4. Bacterial Detection

Respiratory samples were obtained from patients with CPA using noninvasive methods (sputum collection, *n* = 114; 80.3%) or invasive procedures (bronchoscopy, *n* = 28; 19.7%), depending on the patient's underlying comorbidities and decline in lung function. All specimens were examined for bacterial pathogens using Gram staining and inoculated on BAP, MacConkey, and chocolate agar. Subsequent bacterial identification was performed using automatic VITEK® 2 (bioMérieux Inc., Hazelwood, MO, USA) diagnostic systems. All specimens were collected from patients with CPA during the study period. The samples were classified as Murray–Washington classification degree IV or V (degree IV, 10–25 epithelial cells and >25 leukocytes; degree V, <10 epithelial cells and >25 leukocytes per low-magnification field (×100)).

### 2.5. Data Collection

Demographic and clinical data including age, sex, body mass index (BMI), comorbidities, respiratory symptoms, chest CT findings, white blood cell (WBC) count, C-reactive protein (CRP) level, albumin level, bacterial results, treatment history, presence of bilateral lung lesions due to CPA, and outcomes were collected from electronic medical records. We conducted regular follow-up assessments at 3–6 months for patients who participated in the study, according to their condition and clinical practice guidelines [1]. “Breathlessness” represents a modified Medical Research Council dyspnea score ≥2, as previously reported [14]. “Bilateral lung lesions” refers to cases with radiological findings compatible with Aspergillus.

### 2.6. Statistical Analysis

Data are presented as medians with interquartile ranges (IQRs) or as numbers with percentages. Continuous variables were compared using the Mann–Whitney *U* test, while categorical variables were compared using Pearson's chi-square test or Fisher's exact test. The mortality rates of pneumonia after CPA diagnosis were estimated using the Kaplan–Meier method, and the log-rank test was used to compare the mortality rates between the two groups (patients with bacteria vs. patients without bacteria). Univariate and multivariate analyses were performed to identify risk factors for pneumonia-related mortality using the Cox proportional hazards model. Variables that reached statistical significance in the univariate analysis were included in the multivariate analysis, where backward selection with *P* < 0.05 for the entry of variables and *P* > 0.10 for the removal of variables was used to derive a final model. The results are presented as hazard ratios (HRs) with 95% confidence intervals (CI). A *P* value of <0.05 was considered significant. Statistical analyses were performed using SPSS for Windows (version 25.0; IBM Corp., 2018, Chicago, NY).

## 3. Results

### 3.1. Baseline Characteristics

The baseline characteristics of the 142 patients are presented in Table 1. The median age of the patients was 67 years; 94 (66.2%) were male, and the median BMI was 19.6 kg/m^2^. On chest CT, paracavitary infiltrates, mycetomas, and bilateral pulmonary lesions due to *Aspergillus* infection were observed in 133 (93.7%), 41 (28.9%), and 37 (26.1%) patients, respectively. The median WBC, CRP, and albumin were 7,955/*μ*L, 11.0 mg/L, and 4.1 g/dL, respectively. All patients tested positive for serum *Aspergillus* precipitin antibody, and the median serum level of *Aspergillus* precipitin antibody was 40 U/mL. The *Aspergillus* species confirmed by fungal culture was found in only seven (4.9%) patients.

Regarding underlying lung conditions, a significantly higher prevalence of bronchiectasis was observed among female patients, while emphysema was more prevalent among male patients (Table 2).

Of the 142 patients with CPA, 59 (41.5%) did not receive antifungal treatments for various reasons determined by the attending physician: mild disease status (*n* = 11), loss of follow-up (*n* = 12), discontinuation due to adverse drug effects (*n* = 10), and other conditions such as PTB or NTM-PD that were prioritized (*n* = 26). Furthermore, among the 83 (58.5%) patients who received itraconazole, only 51 (35.9%) continued treatment for >6 months during the study period.

### 3.2. Microbiological Characteristics

Microbiological characteristics are shown in Table 3. Bacteria were detected in 73 (51.4%) patients, 11 (7.7%) of whom had multiple microbial species. Eleven bacterial species were identified. The most commonly identified bacteria were *Klebsiella* spp. (*n* = 35; 24.6%), followed by *P. aeruginosa* (*n* = 31; 21.8%), *Escherichia coli* (*n* = 6; 4.2%), and *S. aureus* (*n* = 5; 3.5%). The median interval between blood sampling and bacterial culture was 7 days.

A comparison of the clinical characteristics of CPA patients with and without isolated bacterial species is shown in Table 4. The presence of bacteria was associated with significantly older age (69 years vs. 65 years, *P*=0.024), a higher proportion of females (43.8% vs. 23.2%, *P*=0.013), diabetes (32.9% vs. 15.9%, *P*=0.021), and a history of extrathoracic malignancy (11.0% vs. 1.4%, *P*=0.034). Tables 5 and 6 present comparisons of the clinical characteristics of patients with and without *P. aeruginosa* or *Klebsiella* spp. The presence of isolated *P. aeruginosa* was associated with a higher percentage of females (44.8% vs. 27.9%, *P*=0.009) and bronchiectasis (51.6% vs. 27.0%, *P*=0.016) than patients without *P. aeruginosa* isolates (Table 5). No notable differences in clinical characteristics were observed in the presence of *Klebsiella* spp. (Table 6).

### 3.3. Survival

**Figure 2:**
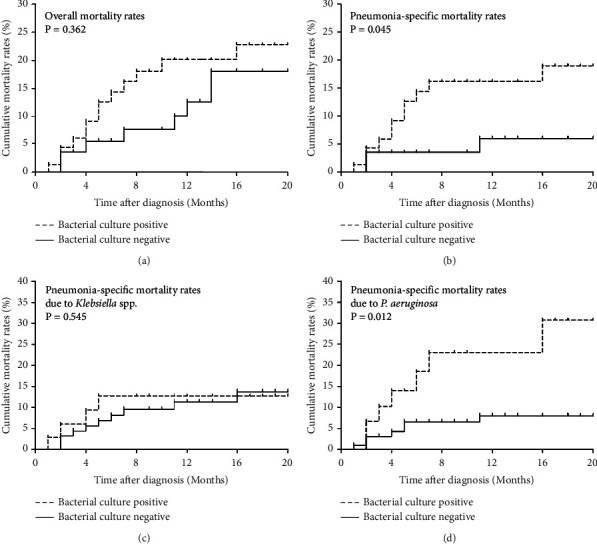
Mortality rates of study participants (*N* = 142). (a) overall mortality rates, (b) pneumonia-specific mortality rates, (c) pneumonia-specific mortality rates due to *Klebsiella* species, and (d) pneumonia-specific mortality rates due to *Pseudomonas aeruginosa*.

Over a median follow-up duration of 11 months (IQR, 4–21 months), the overall survival rate at 1 year was 86%. The survival rate and pneumonia-specific rates in CPA patients with or without bacteria are shown in Figures 2(a) and 2(b). The 1-year mortality rate from pneumonia was 9%, with significantly higher pneumonia-specific mortality rates observed in patients with any bacterial species than in those without it (*P*=0.045). In patients with *Klebsiella* spp. infection, pneumonia-specific mortality rates were similar to those of patients without *Klebsiella* spp. infection (Figure 2(c)). On the contrary, in patients with *P. aeruginosa*, the mortality rate due to pneumonia was significantly higher than in patients without *P. aeruginosa* infection (*P*=0.012) (Figure 2(d)).

Univariate and multivariate Cox proportional hazards regression analyses were performed to evaluate risk factors for pneumonia-related mortality. In the univariate analysis, the presence of *P. aeruginosa* (unadjusted HR, 3.04; *P*=0.018) was significantly associated with pneumonia-related mortality rates (Table 7). Multivariate analysis also revealed that the presence of *P. aeruginosa* (adjusted HR, 3.34; *P*=0.015) was positively associated with pneumonia-related mortality rates (Table 7). The presence of any bacterial species or *Klebsiella* spp. was associated with a higher HR without statistical significance (Tables 8 and 9).

## 4. Discussion

This study aimed to investigate the impact of respiratory bacterial isolation in CPA patients and yielded several significant findings. We examined the prevalence and composition of bacteria in the lower respiratory tract of patients with CPA and found that approximately half of them had bacteria isolated from specimens, including sputum or bronchial washing. Bacterial culture-positive patients were more likely to be older, female, and have diabetes and a history of extrathoracic malignancy than bacterial culture-negative patients. Additionally, bacterial culture positivity was associated with significantly higher pneumonia-specific mortality in patients with CPA. Particularly, *P. aeruginosa* isolation was associated with a significantly increased mortality from pneumonia using the Cox proportional hazards model.

The lungs and respiratory airways contain diverse bacterial communities associated with chronic lung diseases [15]. Excess inflammation in chronic lung disease can also lead to deleterious structural changes in the airway epithelium, which plays a central role in the dynamics of bacteria through various mechanisms, including structural barrier function, mucociliary clearance, and cytokine production [16, 17]. Microbial dysbiosis caused by alterations in bacterial communities can contribute to the exacerbation and progression of chronic lung diseases [18]. Each chronic lung disease exhibits specific patterns of respiratory bacterial composition that can be associated with disease pathogenesis and prognosis [19]. For instance, studies on PTB have described the characteristics of respiratory bacteria [20, 21]. The presence of respiratory bacteria such as *H. influenzae* and *S. pneumoniae* has been associated with a poorer prognosis [20, 22]. In COPD and ILD, *H. influenzae* and *P. aeruginosa* are frequently isolated, and their colonization has been associated with exacerbations or progression [8, 23]. Similarly, the isolation of *P. aeruginosa* has been associated with impaired lung function and increased mortality in bronchiectasis [24]. However, few studies have investigated the clinical impact of bacteria on patients with CPA. Our study revealed that bacteria play a role in the prognosis of CPA, similar to other chronic respiratory diseases.

In our study, we found that half of the CPA patients had bacterial isolation, and the most common species was *K. pneumoniae* followed by *P. aeruginosa,* which is consistent with a previous study in CPA patients that reported *P. aeruginosa* as the most common bacterium [25]. CPA patients with bacteria have characteristics such as older age, diabetes, and a previous history of extrathoracic malignancy, suggesting that these factors can influence host defense and immunity and can contribute to disease progression and mortality [26–28]. Interestingly, we observed a higher rate of bacterial co-occurrence in women, probably because of the higher prevalence of bronchiectasis and NTM-PD.

In this study, the 1-year mortality rate was 14%. Previous research has reported varying 1-year mortality rates, ranging from 7% to 35%, depending on factors such as study period, geographical setting, and sample size [4]. We also found that the pneumonia-specific mortality rate at 1 year was 9%, with a significantly higher mortality rate in patients with positive cultures than in those with negative cultures (*P*=0.045). Dysbiosis within respiratory bacteria can potentially influence host defense and immunity and can affect mortality in culture-positive patients [19]. Furthermore, Aspergillus hyphae release galactosaminogalactan (GAG) during active infection. GAG plays an important role in biofilm formation, induces neutrophil apoptosis, and exerts immunomodulatory effects on host cells [29]. Biofilm interactions between Aspergillus and Pseudomonas are associated with poor clinical outcomes in patients with chronic lung disease [30]. Our study highlights the clinical significance of chronic Pseudomonas infection and underscores the need for proactive identification of bacterial co-infection or colonization.

Our study has several limitations. First, it was conducted in a single referral hospital and included a relatively small number of patients, which could have prevented the generalization of the results. Second, the low proportion (35.9%) of patients who received sufficient antifungal treatment may have contributed to the poor prognosis observed in this study. However, the results of this study can be applied in real-world clinical settings. Third, there may have been selection bias because not all patients were routinely tested for bacterial culture, and the retrospective nature of the study may have led to some patients with CPA not being identified because diagnostic tests were performed only when CPA was clinically suspected. Lastly, in this study, no precise species identification of cultured *Aspergillus* organisms was performed.

In conclusion, our study revealed a diverse range of bacteria in patients with CPA, and clinical differences were observed between patients with and without isolated bacteria. Moreover, CPA patients with isolated bacteria, especially *P. aeruginosa*, have higher mortality rates due to pneumonia. Performing tests to identify bacteria in the lower respiratory tract of patients with CPA can help predict future mortality due to pneumonia. More research with larger cohorts and a prospective design is warranted to validate and expand on these findings.

## Figures and Tables

**Table 1:** Baseline characteristics of study participants (*N* = 142).

	Total (*N* = 142)
Age (years)	67 (60–74)
Sex (male)	94 (66.2)
Body mass index (kg/m^2^)	19.6 (17.3–22.4)
Underlying lung disease^†^	
Previous history of pulmonary tuberculosis	97 (68.3)
Nontuberculous mycobacterial pulmonary disease	48 (33.8)
Bronchiectasis	46 (32.4)
Emphysema	45 (31.7)
Interstitial lung disease	9 (6.3)
Previous history of thoracic malignancy	8 (5.6)
Other comorbidities^†^	
Diabetes mellitus	35 (24.6)
Congestive heart disease	11 (7.7)
Chronic hepatic insufficiency	9 (6.3)
Chronic renal insufficiency	7 (4.9)
Rheumatic disease	9 (6.3)
Previous history of extrathoracic malignancy	9 (6.3)
Chronic pulmonary symptoms^†^	
Cough	62 (43.7)
Sputum	74 (52.1)
Breathlessness^‡^	57 (40.1)
Hemoptysis	57 (40.1)
Chest computed tomographic findings^†^	
Cavitation	142 (100)
Paracavitary infiltrates	133 (93.7)
Mycetoma	41 (28.9)
Consolidation	12 (8.5)
Bilateral pulmonary lesions	37 (26.1)
Laboratory findings	
White blood cells (*μ*L)	7,955 (6,018−9,913)
C-reactive protein (mg/L)	11.0 (2.3–34.0)
Albumin (g/dL)	4.1 (3.6–4.4)
Microbiological tests	
Positive serum Aspergillus precipitin antibody test	142 (100)
Aspergillus culture	7 (4.9)

Data are presented as the median (interquartile range) or number (%). ^†^Cases are duplicated. ^‡^Breathlessness represents a modified Medical Research Council dyspnea score ≥ 2.

**Table 2:** Underlying lung disease^*∗*^ according to sex among study participants (*N* = 142).

	Sex		*P* value
	Male (*n* = 94)	Female (*n* = 48)	
Previous history of pulmonary tuberculosis	69 (73.4)	28 (58.3)	0.086
Nontuberculous mycobacterial pulmonary disease	27 (28.7)	21 (43.8)	0.092
Bronchiectasis	18 (19.1)	28 (58.3)	**<0.001**
Emphysema	44 (46.8)	1 (2.1)	**<0.001**
Interstitial lung disease	8 (8.5)	1 (2.1)	0.273
Previous history of thoracic malignancy	8 (8.5)	0	0.051

Data are presented as the number (%). ^*∗*^Cases are duplicated. Bold values indicate statistical significance at *P* < 0.05.

**Table 3:** Microbiological characteristics of sputum or bronchial washing fluid among study participants (*N* = 142).

	Total
Patients with detected bacteria	73 (51.4)
Single bacterium	62 (43.7)
Multiple bacterial species	11 (7.7)
Total of isolated bacteria	11
Identification of bacteria^†^	
*Klebsiella* species	35 (24.6)
*Klebsiella pneumoniae* ssp*. pneumoniae*	32 (22.5)
*Klebsiella oxytoca*	3 (2.1)
*Pseudomonas aeruginosa*	31 (21.8)
*Escherichia coli*	6 (4.2)
*Enterobacter* species	3 (2.1)
*Enterobacter cloacae* spp. *cloacae*	2 (1.4)
*Enterobacter aerogenes*	1 (0.7)
*Citrobacter freundii*	1 (0.7)
*Morganella morganii* ssp*. morganii*	1 (0.7)
*Raoultella planticola*	1 (0.7)
*Serratia marcescens*	1 (0.7)
*Acinetobacter baumannii*	1 (0.7)
*Staphylococcus aureus*	5 (3.5)
*Staphylococcus epidermidis*	1 (0.7)

Data are presented as the number (%). ^†^Cases are duplicated.

**Table 4:** Comparison of clinical characteristics with and without any species of bacteria in sputum or bronchial washing fluid among study participants (*N* = 142).

	Any species of bacteria		*P* value
	Yes	No	
	(*n* = 73)	(*n* = 69)	
Age (years)	69 (63–76)	65 (57–73)	**0.024**
Sex (male)	41 (56.2)	53 (76.8)	**0.013**
Body mass index (kg/m^2^)	20.1 (17.9–22.8)	18.9 (16.9–21.5)	0.104
Underlying lung disease^†^			
Previous history of pulmonary tuberculosis	50 (68.5)	47 (68.1)	>0.999
Nontuberculous mycobacterial pulmonary disease	27 (37.0)	21 (30.4)	0.479
Bronchiectasis	28 (38.4)	18 (26.1)	0.151
Emphysema	19 (26.0)	26 (37.7)	0.152
Interstitial lung disease	3 (4.1)	6 (8.7)	0.316
Previous history of thoracic malignancy	4 (5.5)	4 (5.8)	>0.999
Other comorbidities^†^			
Diabetes mellitus	24 (32.9)	11 (15.9)	**0.021**
Congestive heart disease	7 (9.6)	4 (5.8)	0.534
Chronic hepatic insufficiency	4 (5.5)	5 (7.2)	0.740
Chronic renal insufficiency	3 (4.1)	4 (5.8)	0.713
Rheumatic disease	3 (4.1)	6 (8.7)	0.316
Previous history of extrathoracic malignancy	8 (11.0)	1 (1.4)	**0.034**
Chronic pulmonary symptoms^†^			
Cough	36 (49.3)	26 (37.7)	0.179
Sputum	38 (52.1)	36 (52.2)	>0.999
Breathlessness^‡^	24 (32.9)	33 (47.8)	0.087
Hemoptysis	31 (42.5)	26 (37.7)	0.610
Chest computed tomographic findings^†^			
Cavitation	73 (100)	69 (100)	NA
Paracavitary infiltrates	69 (94.5)	64 (92.8)	0.740
Mycetoma	20 (27.4)	21 (30.4)	0.715
Consolidation	3 (4.1)	9 (13.0)	0.072
Bilateral pulmonary lesions	18 (24.7)	19 (27.5)	0.707
Laboratory findings			
White blood cells (*μ*L)	8,130 (6,070−10,030)	7,580 (5,785−9,815)	0.431
C-reactive protein (mg/L)	8.9 (2.9–26.8)	13.8 (1.9–56.2)	0.422
Albumin (g/dL)	4.1 (3.6–4.4)	4.1 (3.5–4.4)	0.967

Data are presented as the median (interquartile range) or number (%). ^†^Cases are duplicated. ^‡^Breathlessness represents a modified Medical Research Council dyspnea score ≥ 2. Bold values indicate statistical significance at *P* < 0.05.

**Table 5:** Comparison of clinical characteristics with and without *P. aeruginosa* in sputum or bronchial washing fluid among study participants (*N* = 142).

	*P. aeruginosa*		*P* value
	Yes	No	
	(*n* = 31)	(*n* = 111)	
Age (years)	69 (65–75)	66 (58–74)	0.176
Sex (male)	14 (45.2)	80 (72.1)	**0.009**
Body mass index (kg/m^2^)	21.5 (19.1–23.4)	19.1 (17.1–22.1)	0.068
Underlying lung disease^*∗*^			
Previous history of pulmonary tuberculosis	20 (64.5)	77 (69.4)	0.664
Nontuberculous mycobacterial pulmonary disease	6 (19.4)	42 (37.8)	0.084
Bronchiectasis	16 (51.6)	30 (27.0)	**0.016**
Emphysema	6 (19.4)	39 (35.1)	0.127
Interstitial lung disease	0	9 (8.1)	0.206
Previous history of thoracic malignancy	3 (9.7)	5 (4.5)	0.372
Other comorbidities^*∗*^			
Diabetes mellitus	10 (32.3)	25 (22.5)	0.345
Congestive heart disease	3 (9.7)	8 (7.2)	0.705
Chronic hepatic insufficiency	2 (6.5)	7 (6.3)	>0.999
Chronic renal insufficiency	2 (6.5)	5 (4.5)	0.647
Rheumatic disease	2 (6.5)	7 (6.3)	>0.999
Previous history of extrathoracic malignancy	3 (9.7)	6 (5.4)	0.410
Chronic pulmonary symptoms^*∗*^			
Cough	16 (51.6)	46 (41.4)	0.413
Sputum	17 (54.8)	57 (51.4)	0.840
Breathlessness^†^	13 (41.9)	44 (39.6)	0.838
Hemoptysis	14 (45.2)	43 (38.7)	0.540
Chest computed tomographic findings^*∗*^			
Cavitation	31 (100)	111 (100)	NA
Paracavitary infiltrates	30 (96.8)	103 (92.8)	0.684
Mycetoma	8 (25.8)	33 (29.7)	0.823
Consolidation	2 (6.5)	10 (9.0)	>0.999
Bilateral pulmonary lesions	5 (16.1)	32 (28.8)	0.174
Laboratory findings			
White blood cells (*μ*L)	8,380 (6,310−10,410)	7,650 (5,980−9,740)	0.363
C-reactive protein (mg/L)	15.1 (3.3–27.9)	11.0 (2.3–39.5)	0.912
Albumin (g/dL)	4.0 (3.3–4.3)	4.1 (3.7–4.4)	0.378

Data are presented as the median (interquartile range) or number (%). ^*∗*^Cases are duplicated. ^†^Breathlessness represents a modified Medical Research Council dyspnea score ≥ 2. Bold values indicate statistical significance at *P* < 0.05.

**Table 6:** Comparison of clinical characteristics with and without *Klebsiella* spp. in sputum or bronchial washing fluid among study participants (*N* = 142).

	*Klebsiella* spp.		*P* value
	Yes	No	
	(*n* = 35)	(*n* = 107)	
Age (years)	67 (62–75)	66 (59–74)	0.337
Sex (male)	26 (74.3)	68 (63.6)	0.305
Body mass index (kg/m^2^)	20.8 (16.4–23.0)	19.4 (17.3–21.8)	0.567
Underlying lung disease^*∗*^			
Previous history of pulmonary tuberculosis	26 (74.3)	71 (66.4)	0.412
Nontuberculous mycobacterial pulmonary disease	13 (37.1)	35 (32.7)	0.683
Bronchiectasis	11 (31.4)	35 (32.7)	>0.999
Emphysema	9 (25.7)	36 (33.6)	0.412
Interstitial lung disease	3 (8.6)	6 (5.6)	0.689
Previous history of thoracic malignancy	1 (2.9)	7 (6.5)	0.679
Other comorbidities^*∗*^			
Diabetes mellitus	12 (34.3)	23 (21.5)	0.174
Congestive heart disease	2 (5.7)	9 (8.4)	>0.999
Chronic hepatic insufficiency	3 (8.6)	6 (5.6)	0.689
Chronic renal insufficiency	2 (5.7)	5 (4.7)	>0.999
Rheumatic disease	1 (2.9)	8 (7.5)	0.452
Previous history of extrathoracic malignancy	3 (8.6)	6 (5.6)	0.689
Chronic pulmonary symptoms^*∗*^			
Cough	16 (45.7)	46 (43.0)	0.845
Sputum	17 (48.6)	57 (53.3)	0.698
Breathlessness^†^	11 (31.4)	46 (43.0)	0.241
Hemoptysis	17 (48.6)	40 (37.4)	0.321
Chest computed tomographic findings^*∗*^			
Cavitation	35 (100)	107 (100)	NA
Paracavitary infiltrates	32 (91.4)	101 (94.4)	0.689
Mycetoma	12 (34.3)	29 (27.1)	0.519
Consolidation	2 (5.7)	10 (9.3)	0.730
Bilateral pulmonary lesions	10 (28.6)	27 (25.2)	0.825
Laboratory findings			
White blood cells (*μ*L)	7,520 (5,750−9,920)	7,960 (6,030−9,945)	0.815
C-reactive protein (mg/L)	15.7 (3.1–30.8)	10.4 (2.1–36.7)	0.759
Albumin (g/dL)	4.0 (3.5–4.3)	4.1 (3.7–4.4)	0.291

Data are presented as the median (interquartile range) or number (%). ^*∗*^Cases are duplicated. ^†^Breathlessness represents a modified Medical Research Council dyspnea score ≥ 2.

**Table 7:** Cox regression analysis of pneumonia-specific mortality according to *Pseudomonas aeruginosa* in study participants (*N* = 142).

Variable	Univariate analysis		Multivariate analysis	
	Crude HR (95% CI)	*P* value	Adjusted HR (95% CI)	*P* value
Sex (male)	1.05 (0.40–2.77)	0.924	1.24 (0.43–3.54)	0.694
Age ≥70 years	2.11 (0.84–5.27)	0.111	2.10 (0.80–5.53)	0.132
Diabetes mellitus	1.51 (0.57–4.00)	0.411		
Previous history of extrathoracic malignancy	1.61 (0.37–6.97)	0.527		
Presence of *Pseudomonas aeruginosa*	3.04 (1.21–7.62)	**0.018**	3.34 (1.27–8.80)	**0.015**

Data are presented as hazard ratios (HRs) with 95% confidence intervals. HRs, hazard ratios; CI, confidence intervals. Bold values indicate statistical significance at *P* < 0.05.

**Table 8:** Cox regression analysis of pneumonia-specific mortality according to any species of bacteria in study participants (*N* = 142).

Variable	Univariate analysis		Multivariate analysis	
	Crude HR (95% CI)	*P* value	Adjusted HR (95% CI)	*P* value
Sex (male)	1.05 (0.40–2.77)	0.924		
Age ≥ 70 years	2.11 (0.84–5.27)	0.111	1.90 (0.76–4.77)	0.172
Diabetes mellitus	1.51 (0.57–4.00)	0.411		
Previous history of extrathoracic malignancy	1.61 (0.37–6.97)	0.527		
Presence of any species of bacteria	2.71 (0.98–7.55)	0.056	2.53 (0.90–7.07)	0.077

Data are presented as hazard ratios (HRs) with 95% confidence intervals. HRs, hazard ratios; CI, confidence intervals.

**Table 9:** Cox regression analysis of pneumonia-specific mortality according to *Klebsiella* spp. in study participants (*N* = 142).

Variable	Univariate analysis		Multivariate analysis	
	Crude HR (95% CI)	*P* value	Adjusted HR (95% CI)	*P* value
Sex (male)	1.05 (0.40–2.77)	0.924		
Age ≥70 years	2.11 (0.84–5.27)	0.111	2.04 (0.81–5.13)	0.131
Diabetes mellitus	1.51 (0.57–4.00)	0.411	1.36 (0.51–3.63)	0.536
Previous history of extrathoracic malignancy	1.61 (0.37–6.97)	0.527		
Presence of *Klebsiella* spp.	1.35 (0.51–3.55)	0.549	1.31 (0.49–3.46)	0.592

Data are presented as hazard ratios (HRs) with 95% confidence intervals. HRs, hazard ratios; CI, confidence intervals.

## Data Availability

The data used to support the findings of this study are available from the corresponding author upon reasonable request.

## Abbreviations

BMI: Body mass index
CI: Confidence intervals
COPD: Chronic obstructive pulmonary disease
CPA: Chronic pulmonary aspergillosis
CRP: C-reactive protein
CT: Computed tomography
GAG: Galactosaminogalactan
HRs: Hazard ratios
IgG: Immunoglobulin G
ILD: Interstitial lung disease
IQR: Interquartile range
NTM-PD: Nontuberculous mycobacterial pulmonary disease
PTB: Pulmonary tuberculosis
WBC: White blood cell.
